# Dietary diversity and depression: cross-sectional and longitudinal analyses in Spanish adult population with metabolic syndrome. Findings from PREDIMED-Plus trial

**DOI:** 10.1017/S1368980022001525

**Published:** 2023-03

**Authors:** Naomi Cano-Ibáñez, Lluis Serra-Majem, Sandra Martín-Peláez, Miguel Ángel Martínez-González, Jordi Salas-Salvadó, Dolores Corella, Camille Lassale, Jose Alfredo Martínez, Ángel M Alonso-Gómez, Julia Wärnberg, Jesús Vioque, Dora Romaguera, José López-Miranda, Ramon Estruch, Ana María Gómez-Pérez, José Lapetra, Fernando Fernández-Aranda, Aurora Bueno-Cavanillas, Josep A Tur, Naiara Cubelos, Xavier Pintó, José Juan Gaforio, Pilar Matía-Martín, Josep Vidal, Cristina Calderón, Lidia Daimiel, Emilio Ros, Alfredo Gea, Nancy Babio, Ignacio Manuel Gimenez-Alba, María Dolores Zomeño-Fajardo, Itziar Abete, Lucas Tojal Sierra, Rita P Romero-Galisteo, Manoli García de la Hera, Marian Martín-Padillo, Antonio García-Ríos, Rosa M Casas, JC Fernández-García, José Manuel Santos-Lozano, Estefanía Toledo, Nerea Becerra-Tomas, Jose V Sorli, Helmut Schröder, María A Zulet, Carolina Sorto-Sánchez, Javier Diez-Espino, Carlos Gómez-Martínez, Montse Fitó, Almudena Sánchez-Villegas

**Affiliations:** 1 Department of Preventive Medicine and Public Health, Faculty of Medicine, University of Granada, Avda. De la Investigación, 11, Granada, 18016, Spain; 2 Centro de Investigación Biomédica en Red Epidemiología y Salud Pública (CIBERESP), Institute of Health Carlos III, Madrid, Spain; 3 Instituto de Investigación Biosanitaria de Granada (ibs.GRANADA), Granada, Spain; 4 Consorcio CIBER, M.P. Fisiopatología de la Obesidad y Nutrición (CIBERObn), Instituto de Salud Carlos III (ISCIII), Madrid, Spain; 5 Research Institute of Biomedical and Health Sciences (IUIBS), University of Las Palmas de Gran Canaria, Las Palmas de Gran Canaria, Spain; 6 Department of Preventive Medicine and Public Health, IDISNA, University of Navarre, Pamplona, Spain; 7 Department of Nutrition, Harvard T.H. Chan School of Public Health, Boston, MA, USA; 8 Universitat Rovira i Virgili, Departament de Bioquímica i Biotecnologia, Unitat de Nutrició Humana, Reus, Spain; 9 University Hospital of Sant Joan de Reus, Nutrition Unit, Reus, Spain; 10 Institut d’Investigació Sanitària Pere Virgili (IISPV), Reus, Spain; 11 Department of Preventive Medicine, University of Valencia, Valencia, Spain; 12 Unit of Cardiovascular Risk and Nutrition, Institut Hospital del mar de Investigaciones Médicas Municipal d’Investigació Médica (IMIM), Barcelona, Spain; 13 Department of Nutrition, Food Sciences and Physiology, Center for Nutrition Research, University of Navarra, Pamplona, Spain; 14 Cardiometabolic Nutrition Group, IMDEA Food, CEI UAM + CSIC, Madrid, Spain; 15 Bioaraba Health Research Institute, Cardiovascular, Respiratory and Metabolic Area; Osakidetza Basque Health Service, Araba University Hospital, University of the Basque Country UPV/EHU, Vitoria-Gasteiz, Spain; 16 Department of Nursing, School of Health Sciences, University of Malaga, Instituto de Investigación Biomédica de Málaga (IBIMA), Málaga, Spain; 17 Nutritional Epidemiology Unit, Instituto de Investigación Sanitaria y Biomédica de Alicante, Universidad Miguel Hernández (ISABIAL-UMH), Alicante, Spain; 18 Health Research Institute of the Balearic Islands (IdISBa), Palma de Mallorca, Spain; 19 Lipids and Atherosclerosis Unit, Department of Internal Medicine, Maimonides Biomedical Research Institute of Córdoba (IMIBIC), Reina Sofía University Hospital, University of Córdoba, Córdoba, Spain; 20 Department of Internal Medicine, Institut dÌnvestigacions Biomèdiques August Pi Sunyer (IDIBAPS), Hospital Clinic, University of Barcelona, Barcelona, Spain; 21 Virgen de la Victoria Hospital, Department of Endocrinology, Instituto de Investigación Biomédica de Málga (IBIMA), University of Málaga, Málaga, Spain; 22 Department of Family Medicine, Research Unit, Distrito Sanitario Atención Primaria Sevilla, Sevilla, Spain; 23 Research Group on Community Nutrition & Oxidative Stress, University of Balearic Islands, Palma de Mallorca, Spain; 24 José Aguado Health Centre, Institute of Biomedicine (IBIOMED), University of León, León, Spain; 25 Lipids and Vascular Risk Unit, Internal Medicine, Hospital Universitario de Bellvitge, Hospitalet de Llobregat, Barcelona, Spain; 26 Center for Advanced Studies in Olive Grove and Olive Oils, University of Jaén, Jaén, Spain; 27 Department of Endocrinology and Nutrition, Instituto de Investigación Sanitaria Hospital Clínico San Carlos (IdISSC), Madrid, Spain; 28 CIBER Diabetes y Enfermedades Metabólicas (CIBERDEM), Instituto de Salud Carlos III (ISCIII), Madrid, Spain; 29 Department of Endocrinology, Institut dÌnvestigacions Biomèdiques August Pi Sunyer (IDIBAPS), Hospital Clinic, University of Barcelona, Barcelona, Spain; 30 Department of Endocrinology and Nutrition, Hospital Fundación Jiménez-Díaz, Instituto de Investigaciones Biomédicas IISFJD, University Autónoma, Madrid, Spain; 31 Nutritional Control of the Epigenome Group, Precision Nutrition and Obesity Program, IMDEA Food, CEI UAM + CSIC, Madrid, Spain; 32 Nutritional Epidemiology Unit, Miguel Hernández University, ISABIAL-FISABIO, Alicante, Spain; 33 Servicio Navarro de Salud-Osasunbidea-Instituto de Investigación Sanitaria de Navarra (IdiSNA), Pamplona, Navarra, Spain

**Keywords:** Dietary diversity score, Depression, PREDIMED-Plus study

## Abstract

**Objective:**

To examine the cross-sectional and longitudinal (2-year follow-up) associations between dietary diversity (DD) and depressive symptoms.

**Design:**

An energy-adjusted dietary diversity score (DDS) was assessed using a validated FFQ and was categorised into quartiles (Q). The variety in each food group was classified into four categories of diversity (C). Depressive symptoms were assessed with Beck Depression Inventory-II (Beck II) questionnaire and depression cases defined as physician-diagnosed or Beck II >= 18. Linear and logistic regression models were used.

**Setting:**

Spanish older adults with metabolic syndrome (MetS).

**Participants:**

A total of 6625 adults aged 55–75 years from the PREDIMED-Plus study with overweight or obesity and MetS.

**Results:**

Total DDS was inversely and statistically significantly associated with depression in the cross-sectional analysis conducted; OR Q4 *v*. Q1 = 0·76 (95 % CI (0·64, 0·90)). This was driven by high diversity compared to low diversity (C3 *v*. C1) of vegetables (OR = 0·75, 95 % CI (0·57, 0·93)), cereals (OR = 0·72 (95 % CI (0·56, 0·94)) and proteins (OR = 0·27, 95 % CI (0·11, 0·62)). In the longitudinal analysis, there was no significant association between the baseline DDS and changes in depressive symptoms after 2 years of follow-up, except for DD in vegetables C4 *v*. C1 = (*β* = 0·70, 95 % CI (0·05, 1·35)).

**Conclusions:**

According to our results, DD is inversely associated with depressive symptoms, but eating more diverse does not seem to reduce the risk of future depression. Additional longitudinal studies (with longer follow-up) are needed to confirm these findings.

The metabolic syndrome (MetS) is defined as a group of metabolic abnormalities that include central obesity, insulin resistance, dyslipidaemia and hypertension, which are risk factors for the development of CVD^(1)^. In addition, this metabolic alteration has been associated with an increased risk of developing other chronic diseases as cancer^(2)^, neurodegenerative diseases^(3)^ and mental disorders, such as depression^(4)^. Depression is a common mental disorder, particularly in older adults^(5)^, being the third largest cause of years lived with disability in developed countries.

Some authors have pointed out that the modification of lifestyle factors, including inactivity and unhealthy dietary intake, could prevent and manage the progression of depression^(6)^. However, the most common treatments for depressive symptoms in late life is the use of antidepressive medications and psychotherapy, which are not effective in some patients and are a burden on health care utilisation and costs^(7)^.

Regarding the relationship between diet and depression, several studies point out towards a bidirectional association, with the possibility of a reverse causality between them. On the one hand, subjects with depression have worse dietary habits^(8)^ and on the other hand, healthy dietary patterns have been shown to be beneficial reducing the risk of depressive outcomes^(9)^. Hence, healthy dietary patterns have been shown to be beneficial reducing the risk of depressive outcomes. One possible explanation is that dietary quality might modulate several brain pathways including low-grade inflammation and oxidative stress, which intervene in the aetiology of depression^(10)^. Among the different dietary patterns, the strongest evidence for a reduced risk of depression have been found for Mediterranean diet. This fact could be explained by the high diversity of healthy food groups that characterises this dietary pattern, increasing the likelihood to meet nutritional requirements^(11)^. Despite of this, a recent meta-analysis have analysed a subset of studies that controlled for baseline symptoms of depression, reporting no association between diet quality and depression risk^(12)^. So, clear inconsistencies in establishing the diet–depression link still exist.

Dietary diversity (DD) has been universally identified as a key element of high-quality diets. The dietary diversity score (DDS) is a simple count of food groups consumed, in conformity with advices provided by dietary guidelines as indicators of nutritional adequacy worldwide. In patients with mood disorders, particularly prenatal and postpartum women^(13)^, and in younger adult population^(14)^ deficiencies have been found, for nutrients including Ca, vitamins B_9_, B_12_ and *n*-3 fatty acids.

DDS, an useful indicator of nutrient adequacy, has been found to be inversely associated with anxiety after adjusting for socio-economic and lifestyle factors^(15)^. International dietary recommendations in general, and the Spanish dietary guidelines in particular, promote a healthy diet to reduce the incidence of diet-related chronic diseases. The healthy message that the Spanish Society of Community Nutrition (SENC) conveys to the population is that ‘Diet should be balanced, moderate and varied’^(16)^. Meanwhile, the role of a varied diet over chronic diseases as obesity^(17)^, cancer^(18)^ or CVD^(19)^ has been adressed, specifically the potential prevention of depression is yet to be determined. Understanding and addressing the possible role of DD in depressive symptoms can be of great public health importance.

To our knowledge, no previous study has focused on the relationship between the DD and mental health among older Spanish population with MetS. Hence, our research was designed to examine the cross-sectional and longitudinal (2-year follow-up) associations between DD and depressive symptoms in a cohort of Spanish older adults with MetS.

## Methods

### Design of the study

The PREDIMED-Plus study is a randomised primary prevention trial involving twenty-two centres throughout Spain with a planned follow-up of 6 years. Participants were randomly assigned to two groups: intervention group and control group. The main objective of the clinical trial is to determine the effect on cardiovascular mortality of an intensive dietary advice for weight loss based on a traditional hypocaloric Mediterranean dietary pattern promoting physical activity and behavioural therapy (intervention group) *v*. Mediterranean-type dietary advice for CVD prevention in the context of usual health care (control group). More detailed information on the study protocol can be found in the publication by Martínez-González *et al.* ^(20)^. The database used was updated on 26 June 2020.

### Ethics approval

The trial was registered at the International Standard Randomized Controlled Trial (ISRCTN: http://www.isrctn.com/ISRCTN89898870) with number 89898870 and registration date of 24 July 2014. All participants gave written informed consent, and the study was approved by the Research Ethics Committees from all recruitment centres, according to the ethical standards of the Declaration of Helsinki.

### Participants and data collection procedures

Eligible participants were men (aged 55–75 years) and women (aged 60–75 years), with overweight or obesity (BMI ≥ 27 and <40 kg/m^2^), who at baseline met at least three components of the MetS: TAG level ≥150 mg/dl, blood glucose ≥ 100 mg/dl or use of oral antidiabetic drugs, systolic blood pressure ≥130 mmHg and diastolic blood pressure ≥85 mmHg or use of antihypertensive drugs and/or HDL-cholesterol level <40 mg/dl for men and <50 mg/dl for women according to the harmonised criteria of the International Diabetes Federation and the American Heart Association and National Heart, Lung and Blood Institute^(21)^ and without other neurological or endocrine disease active.

**Fig. 1:**
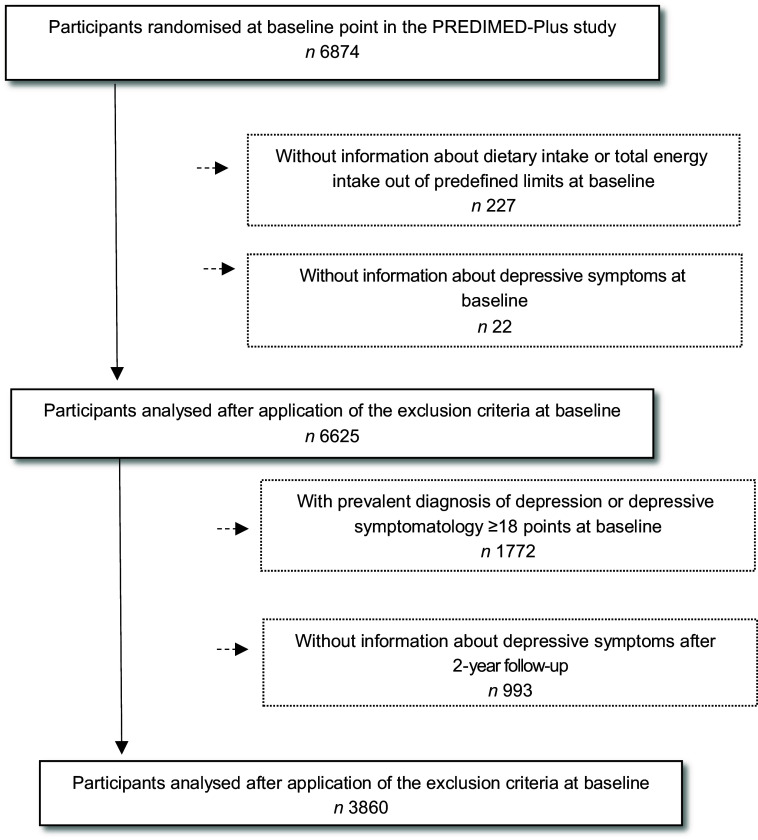
Flow chart of the study participants

Of the 6874 participants enrolled in the PREDIMED-Plus study, only participants who completed a semi-quantitative FFQ and a depressive symptoms questionnaire (Beck Depression Inventory-II, Beck II) at baseline were included in the current analysis. Those participants with missing dietary data and with extreme energy intakes (<500 or >3500 kcal/d for women and <800 or >4000 kcal/d for men)^(22)^ (*n* 227) at baseline were excluded. Among the available participants, we also excluded those who failed to complete the Beck II questionnaire at baseline (*n* 22). The final sample for the cross-sectional analysis was 6625 participants. For the longitudinal analysis, out of the eligible individuals, we excluded those with prevalent depression at baseline, those who had a Beck II score ≥18 points at baseline (*n* 1772), and those who did not complete the Beck II questionnaire after 2 years of follow-up (*n* 993). Finally, for the longitudinal analysis, 3860 participants were included (Fig. 1).

### Dietary intake assessment

At baseline, trained dieticians filled out a validated 143-item semi-quantitative FFQ^(23)^ in a face-to-face interview. The FFQ provides a list of foods commonly used by the Spanish population and asks about the consumption of these foods during the previous year. From this questionnaire, total energy and nutrient intake were calculated based on Spanish food composition tables^(24,25)^.

### Dietary diversity score construction

The 143-item FFQ was also used to calculate an energy-adjusted DD score (DDS). This DDS was calculated by the method originally developed by Kant *et al.* ^(26)^ and recently reported by Farhangi *et al.* ^(27)^ and Cano-Ibáñez *et al.* ^(11,18,28)^. DDS was calculated based on the method using the food groups recommended by the Spanish guidelines’ pyramid^(16)^. Table 1 shows a detailed description of food groups and subgroups considered in the DDS and their recommended consumption measured as servings/d.

**Table 1:** Food groups and the recommended servings/d/week used in the dietary diversity score (DDS) according to the Spanish guidelines

Food groups	Food subgroups	Recommended servings
Vegetables	(1) Green vegetables: spinach, cruciferous, lettuce, green beans, eggplant, peppers and asparagus (2) Tomatoes (3) Yellow vegetables: carrots and pumpkin (4) Mushrooms	2 servings/d
Fruits	(1) Citrus fruits: orange (2) Tropical Fruits: banana, kiwi and grapes (3) Other seasonal fruits: Apple, peach, strawberries, watermelon and melon	3 servings/d
Dairy products	(1) Milk: low fat and high fat (2) Yogurt: low fat and high fat (3) Cheese: low fat and high fat	2 servings/d
Cereals	(1) Potatoes (2) Grain: bread, pasta, rice, and whole breakfast cereals	4 servings/d
Protein food groups	(1) Legumes: peas, beans, lentils and chickpeas (2) White meats: poultry and rabbit (3) Fish: oily fish, white fish and other shellfish/seafood (4) Eggs (5) Nuts: almonds, pistachios and walnuts	3 servings/week

The non-recommended food groups (which should be consumed only exceptionally)^(29)^ have not been included in the calculation of the DD. These are products with low nutritional content and unhealthy and, therefore, their variety is not desirable. These food categories include those foods containing refined sugars and alcohol (bakery products, ice cream, pastries, sweetened beverages, chocolate, fruit-flavoured drinks and alcohol beverages) and food groups high in salt, cholesterol and/or *trans*-fat and saturated fat (butter, cream, fried foods, unhealthy vegetable fats, processed meats, sauces, ready meals, condiments and snacks). Therefore, we only analysed diversity of recommended food groups^(30)^. To be counted as a consumer for any of the food group categories reported previously, the participant should consume at least one-half of the recommended serving per d for each of the items included in the food group, scoring with 2 points for each item. A maximum score of 2 was awarded to each of the five groups and so that each participant received a score ranging from 0 (minimum) to 10 (maximum). To calculate the score of each group, the number of subgroups consumed was divided by the total number of subgroups in each main group, and then it was multiplied by 2. The sum of the scores of the five main groups is reported as the total score. The score was adjusted for total energy intake according to the residual method proposed by Willett *et al.* ^(22)^, due to the general concern that high food variety might be a consequence of overconsumption of energy^(31)^. For example, if the Spanish nutritional recommendation advises a usual vegetable intake of two servings per d, for each vegetable item, participants should consume at least one serving/d). Thus, if the consumption per d for a vegetable item is lower than one serving, the value for this item will be 0; conversely, if the consumption is higher than one serving, the value will be 2. For the five considered groups, the procedure is similar. Finally, DDS was categorised in quartiles (Q) and the cut-off points were 3·9, 4·6, 5·4 and 8·0. The variety in each food group was classified into four categories (C) (C1 = 0 points), (C2 => 0–≤0·5 points), (C3 => 0·5–<1 points) and (C4 ≥ 1 point).

### Outcome assessment

Depressive symptoms were collected at baseline and at 1 and 2 years of follow-up visits by trained PREDIMED-Plus staff through a validated questionnaire, the Beck-II. The Beck II includes twenty-one multiple-choice questions, rating on a scale of 0 to 3 according to symptom severity. Total score of the Beck-II questionnaire ranges from 0 to 63 points^(32)^. Prevalent depression was defined as the presence of depressive symptoms at baseline (Beck-II ≥ 18 points) or a current depression diagnosis. The depression diagnosis was collected at baseline, and it was defined as a self-reported lifetime medical diagnosis of depression. Changes in depressive symptomatology were calculated as the difference in Beck-II questionnaire score between the baseline and the 2-year score.

### Covariate assessment

At baseline and 1-year of follow-up visits, participants filled out a general questionnaire to provide data on lifestyle habits and socio-economic factors. Sociodemographic and lifestyle variables were categorised as follows: educational level (three categories: primary level, secondary level and tertiary level which includes university studies), civil status (two categories: married or not, which includes widowed, divorced/singled or others) and whether participants lived alone or not. Other lifestyle variables such as smoking habits (three categories: smoker, never smoker and current smoker), leisure-physical activity status (three categories: active, moderately active and less active) and sleep duration (h/d) were also recorded. Regarding the hours of sleep, participants reported both the average amount on weekdays and weekends. The non-validated and open question used was: ‘How many hours do you sleep on average per d on weekdays and weekends?’ Leisure-time physical activity was measured by the short form of the Minnesota Leisure Time Physical Activity Questionnaire validated for the Spanish population^(33,34)^. Leisure-time activities were computed by assigning a metabolic equivalent score to each activity, multiplied by the time spent for each activity and summing up all activities. Time spent and intensity in leisure-physical activity was calculated as a product of the frequency and duration of six types of activities categorised into three intensities: light PA (< 4 Metabolic Equivalent Tasks (MET)) – walking at a slow/normal pace; moderate PA (4–5·5 MET) – brisk walking and gardening; and vigorous PA (≥ 6·0 MET) – walking in the countryside, climbing stairs, exercise or playing sports.^(35)^. Anthropometric parameters were measured in every follow-up visit according to the PREDIMED-Plus protocol. The measures collected were height (using a wall-mounted stadiometer, in m^2^) and weight (using high-quality electronic calibrated scales, in kg). BMI was calculated as weight in kilograms by the square of height in metres (kg/m^2^). Finally, personal history of baseline chronic diseases (hypertension, dyslipidemia and type 2 diabetes) was collected from the patients’ medical records.

### Statistical analysis

Statistical analyses were performed using STATA software (version 15.0, StataCorp., LP). For the current study, we used the PREDIMED-Plus longitudinal database generated on 26 June 2020 (202006290731_PREDIMEDplus). Data are presented as mean and standard deviations for continuous variables or number and percentages for categorical variables. Cut of points for DDS were defined by quartiles (Q1, low diversity intake and Q4, high diversity intake). Cut of points for each food groups were defined by categories (C1, low diversity intake and C4, high diversity intake).

#### Performance of cross-sectional analysis

Logistic regression models were fitted to assess the relationship between the energy-adjusted total DDS and each of the food groups and the prevalence of depression at baseline (cross-sectional analysis). OR and their 95 % CI were calculated considering the lowest quartile as the reference category. All cross-sectional analyses were adjusted for potential confounders based on prior knowledge: sex, age, smoking habits, physical activity, educational level, BMI, living alone, civil status, sleep duration, presence of chronic diseases, allocation group and recruitment centre. Moreover, in order to assess the effect of diet quality over depressive symptomatology at baseline, we performed an ancillary analyses, excluding all depression cases in which age of depression diagnosis was not available or in which the diagnosis date was very remote (more than 10 years before enrolment) (*n* 1378). These data were obtained through medical records.

#### Performance of longitudinal analysis

The association between the baseline and their changes was evaluated through multivariable regression models adjusted for the same potential confounders mentioned above plus depressive symptomatology at baseline. We also analysed the possible interaction between DDS and allocation group (intervention and control group). Regression coefficients (*β*) and their 95 % CI were calculated. Finally, the exclusion of individuals with high baseline depressive symptomology could limit the possibility of finding longitudinal associations. For this reason, we performed an ancillary analysis not excluding those subjects with a Beck-II score higher than 18 points at baseline or with prevalent depression diagnosis at baseline. Statistical significance was set at *P* < 0·05.

## Results

### Baseline characteristics of the study participants according to dietary diversity score quartiles

This study analysed a sample of 6625 participants from the PREDIMED-Plus cohort. Table 2 provides an overview of the sample characteristics according to baseline DDS quartiles. There were statistically significant differences in the distribution of sociodemographic and lifestyles characteristics across DDS quartiles. Compared to those in the higher quartile of diversity, participants in the lowest quartile were more likely to be younger, male, current smokers and with higher educational level (tertiary school).

**Table 2:** Baseline characteristics of PREDIMED-Plus participants according to quartiles of DDS (total population = 6625)

	Q1 (*n* 1657)		Q2 (*n* 1656)		Q3 (*n* 1656)		Q4 (*n* 1656)		*P*-value
	*n*	%	*n*	%	*n*	%	*n*	%	
Age (years)									
Mean	64·1		64·8		65·2		65·8		<0·001
sd	5·1		4·9		4·8		4·7		
Sex									
Male	1122	67·7	917	55·4	777	46·9	602	36·4	<0·001
Smoking habits									
Current smoker	297	17·9	198	12·0	173	10·5	153	9·2	<0·001
Former smoker	802	48·4	744	44·9	725	43·8	597	36·1	
Never smoker	549	33·1	708	42·8	753	45·5	898	54·2	
Without information	9	0·5	6	0·4	5	0·3	8	0·5	
Physical activity									
Less active	1023	61·9	992	60·0	989	60·0	949	57·6	0·296
Moderately active	295	17·9	306	18·5	322	19·5	326	19·8	
Active	339	20·3	358	21·5	345	20·6	381	22·6	
Educational level									
Tertiary	423	25·5	364	22·0	342	20·7	325	19·6	<0·001
Secondary	537	32·4	485	29·3	453	27·4	436	26·3	
Primary	697	42·1	807	48·7	861	52·0	895	54·1	
Civil status									
Married	1266	76·7	1260	76·4	1252	75·8	1286	77·8	0·028
Living alone (yes)	194	11·7	189	11·4	219	13·2	214	12·9	0·307
BMI (kg/m^2^)									
Mean	32·6		32·5		32·5		32·5		0·727
sd	3·4		3·4		3·5		3·5		
Presence of diseases									
Hypercholesterolemia	1137	68·6	1157	69·9	1144	69·1	1150	69·4	0·412
Type 2 diabetes	440	26·6	445	26·9	468	28·3	470	28·4	0·598
Hypertension	1382	83·4	1375	83·0	1406	84·9	1362	82·3	0·229
*Prevalence of depressive symptoms	424	25·6	437	26·4	462	27·9	449	27·1	0·479
Baseline score of Beck									
Mean	8·2		8·2		8·5		9·0		0·005
sd	7·4		7·4		7·5		7·5		
2-year score of Beck									
Mean	6·5		6·5		6·8		7·2		0·020
sd	7·0		6·8		7·1		7·0		

* Prevalence of depressive symptoms: prevalence of depressive symptoms was defined as the presence of depressive symptoms at baseline (Beck≥18 points) or a current depression diagnosis. DDS cut-off points for each quartile: (Q1 = 0.8–3.9, Q2 = 4.0–4.6, Q3 = 4.7–5.4 and Q4 = 5.5–8.0).

Values are presented as means ± sd for continuous variables and *n* (%) for categorical variables.

Pearson’s chi-square test was performed for categorical variables and ANOVA test for continuous variables (Q1, less diversity; Q4, more diversity).

### Cross-sectional associations between dietary diversity score and variety in food intake and depressive symptomatology (assessed by Beck-II score at baseline point)

As seen in Table 3, total DDS was not associated with depressive symptomology (assessed by Beck-II score) at baseline. Considering each of the components of the total DDS separately, we found significant associations between the consumption of high diversity of groups of vegetables and depressive symptoms compared to the lowest diversity category: *β*-coefficients (95 % CI) for successive categories (C2–C4 *v*. C1) were −0·86 (−1·58, −0·15); −0·81 (−1·47, −0·14) and −0·69 (−1·37, −0·01), respectively.

**Table 3:** Multivariable linear regression models for the association between total DDS and variety in food intake and Beck Depression Inventory-II score at baseline in the PREDIMED-Plus study participants. *β*-Coefficients (95 % confidence intervals) (total population = 6625)

Total DDS	Q1 (*n* 1657)		Q2 (*n* 1656)		Q3 (*n* 1656)		Q4 (*n* 1656)		*P*_for trend_
	*β*	95 % CI	*β*	95 % CI	*β*	95 % CI	*β*	95 % CI	
Model 1	0	Ref.	−0·51	−1·00, −0·01	−0·50	−1·00, 0·02	−0·33	−0·84, 0·18	0·231
Model 2	0	Ref.	−0·45	−0·95, 0·04	−0·46	−0·96, 0·04	−0·24	−0·75, 0·26	0·375
Vegetable group	C1 (*n* 551)		C2 (*n* 1319)		C3 (*n* 2492)		C4 (*n* 2263)		
Model 1	0	Ref.	−0·89	−1·61, −0·17	−0·85	−1·52, −0·18	−0·63	−1·31, 0·05	0·506
Model 2	0	Ref.	−0·86	−1·58, −0·15	−0·81	−1·47, −0·14	−0·69	−1·37, −0·01	0·335
Fruit group	C1 (*n* 848)		C2 (*n* 4529)		C3 (*n* 779)		C4 (*n* 469)		
Model 1	0	Ref.	−0·27	−0·80, 0·26	−0·26	−0·97, 0·45	−0·48	−1·30, 0·35	0·312
Model 2	0	Ref.	−0·25	−0·78, 0·29	−0·32	−1·03, 0·40	−0·72	−1·55, 0·11	0·104
Cereal group	C1 (*n* 353)		C2 (*n* 4791)		C3 (*n* 1396)		C4 (*n* 85)		
Model 1	0	Ref.	−0·04	−0·82, 0·75	0·32	−0·52, 1·17	0·52	−1·20, 2·24	0·123
Model 2	0	Ref.	−0·41	−1·21, 0·38	−0·16	−1·04, 0·73	0·30	−1·46, 2·07	0·464
Proteins group	C1 (*n* 25)		C2 (*n* 1258)		C3 (*n* 2787)		C4 (*n* 2555)		
Model 1	0	Ref.	−1·79	−4·67, 1·08	−2·03	−4·89, 0·83	−2·03	−4·89, 0·82	0·282
Model 2	0	Ref.	−1·83	−4·67, 1·00	−2·15	−4·98, 0·67	−2·24	−5·07, 0·59	0·095
Dairy group	C1 (*n* 690)		C2 (*n* 2454)		C3 (*n* 2622)		C4 (*n* 859)		
Model 1	0	Ref.	0·49	−0·12, 1·10	0·40	−0·21, 1·01	0·55	−0·18, 1·28	0·327
Model 2	0	Ref.	0·36	−0·25, 0·97	0·32	−0·30, 0·93	0·29	−0·45, 1·03	0·638

C, category; DDS, dietary diversity score; Q, quartile (Q1, less diversity; Q4, more diversity).

Values are presented as *β*-coefficients and 95 % CI for Beck Depression Inventory-II score at baseline as continuous variable according to total DDS and variety in food intake.

Model 1: Adjusted for sex and age.

Model 2: Additionally adjusted for energy intake, smoking habits, physical activity, educational level, BMI, living alone, civil status, sleep duration and presence of chronic diseases.

Values presented in bald showed a statistically significant association (*P* < 0·05).

DDS cut-off points for each quartile: (Q1 = 0·8–3·9, Q2 = 4·0–4·6, Q3 = 4·7–5·4 and Q4 = 5·5–8·0).

The variety in each food group was classified into four categories (C): (C1 = 0 points), (C2 => 0–≤0·5 points), (C3 => 0·5–<1 points) and (C4 ≥ 1 point).

### Cross-sectional associations between dietary diversity score and variety in food intake and prevalence of depression

Total DDS was inversely and significantly associated with prevalence of depression in logistic analysis (Table 4). Participants in the highest quartile of total DDS showed lower odds of depression as compared to those participants in the lowest quartile (OR = 0·76, 95 % CI (0·64, 0·90)). Regarding the specific components of the total DDS, high (C3) or very high (C4) diversity of groups of vegetables reduced the odds of depression (OR = 78, 95 % CI (0·63, 0·97)) and (OR = 0·75, 95 % CI (0·60, 0·94)), respectively. In the case of proteins, the OR (95 % CI) were 0·26 (0·11, 0·61) (C3) and 0·24 (0·10, 0·56) (C4) as compared to the reference category (C1). For cereals, only moderate diversity in intake was associated with lower probability of depression. The OR (95 % CI) for C2 and C3 were 0·69 (0·54, 0·89) and 0·71 (0·54, 0·94), respectively.

**Table 4:** Multivariable logistic regression models for the association between total DDS and variety in food intake and prevalence of depression in the PREDIMED-Plus study participants. Odds ratios (95 % confidence intervals) (total population = 6625)

Total DDS	Q1 (*n* 1657)		Q2 (*n* 1656)		Q3 (*n* 1656)		Q4 (*n* 1656)		*P*_for trend_ *
	OR	95 % CI	OR	95 % CI	OR	95 % CI	OR	95 % CI	
Model 1	1	Ref.	0·89	0·75, 1·04	0·87	0·74,1·02	0·73	0·62, 0·87	<0·001
Model 2	1	Ref.	0·92	0·78, 1·08	0·88	0·75, 1·04	0·76	0·64, 0·90	0·001
Vegetable group	C1 (*n* 551)		C2 (*n* 1319)		C3 (*n* 2492)		C4 (*n* 2263)		
Model 1	1	Ref.	0·82	0·65, 1·04	0·76	0·61, 0·94	0·72	0·58, 0·90	0·004
Model 2	1	Ref.	0·83	0·65, 1·05	0·78	0·63, 0·97	0·75	0·60, 0·94	0·017
Fruit group	C1 (*n* 848)		C2 (*n* 4529)		C3 (*n* 779)		C4 (*n* 469)		
Model 1	1	Ref.	0·89	0·75, 1·06	0·80	0·63, 1·00	0·81	0·62, 1·06	0·051
Model 2	1	Ref.	0·91	0·76, 1·08	0·81	0·64, 1·03	0·79	0·60, 1·04	0·043
Cereal group	C1 (*n* 353)		C2 (*n* 4791)		C3 (*n* 1396)		C4 (*n* 85)		
Model 1	1	Ref.	0·69	0·54, 0·87	0·71	0·55, 0·92	0·72	0·41, 1·25	0·197
Model 2	1	Ref.	0·69	0·54, 0·89	0·71	0·54, 0·94	0·81	0·46, 1·45	0·320
Proteins group	C1 (*n* 25)		C2 (*n* 1258)		C3 (*n* 2787)		C4 (*n* 2555)		
Model 1	1	Ref.	0·31	0·13, 0·71	0·26	0·11, 0·59	0·23	0·10, 0·52	<0·001
Model 2	1	Ref.	0·31	0·13, 0·72	0·26	0·11, 0·61	0·24	0·10, 0·56	<0·001
Dairy group	C1 (*n* 690)		C2 (*n* 2454)		C3 (*n* 2622)		C4 (*n* 859)		
Model 1	1	Ref.	0·97	0·79, 1·19	0·89	0·73, 1·08	0·88	0·69, 1·11	0·105
Model 2	1	Ref.	0·99	0·81, 1·21	0·93	0·76, 1·15	0·91	0·72, 1·17	0·292

C, category; DDS, dietary diversity score; Q, quartile (Q1, less diversity; Q4, more diversity).

* DDS/food group measure as continuous variables in order to estimate *P*_for trend._

Values are presented as OR and 95 % CI for prevalence of depression (≥18 p at Beck Depression Inventory II and/or a current depression diagnosis) as categorical variable according to total DDS and variety in food intake.

Model 1: Adjusted for sex and age.

Model 2: Additionally adjusted for energy intake, smoking habits, physical activity, educational level, BMI, living alone, civil status, sleep duration and presence of chronic diseases.

Values presented in bald showed a statistically significant association (*P* < 0·05).

DDS cut-off points for each quartile: (Q1 = 0·8–3·9, Q2 = 4·0–4·6, Q3 = 4·7–5·4, and Q4 = 5·5–8·0).

The variety in each food group was classified into four categories (C): (C1 = 0 points), (C2 => 0–≤0·5 points), (C3 => 0·5–<1 points) and (C4 ≥ 1 point).

In ancillary analyses performed, we excluded all depression cases in which age of depression diagnosis was not available or in which the diagnosis date was very remote (more than 10 years before enrolment) (*n* 1378). In this subsample (*n* 5247, cases = 394), the results were no longer significant although the magnitude of effect was quite similar to that observed in the overall sample. OR and 95 % CI for successive quartiles of DDS were 1 (ref.), 0·92 (0·68, 1·24), 0·87 (0·64, 1·17) and 0·81 (0·60, 1·10).

### Longitudinal associations between total dietary diversity score and variety in food intake and changes in depressive symptomatology after 2 years of follow-up

The association between total DDS and variety in food intake and changes in depressive symptomatology after 2 years of follow-up is presented in Table 5. We did not find any significant association between total DDS or each of the food groups considered and changes in depressive symptomatology after 2 years of follow-up even after adjustment for potentially confounding factors, except for the vegetable group (*β*-coefficient for C4 = 0·70, 95 % CI (0·05, 1·35)), which, unexpectedly, showed a positive association with an increase of depressive symptomatology over time.

**Table 5:** Change in Beck Depression Inventory-II score across quartiles of DDS and variety in food intake after 2 year of follow-up in the PREDIMED-Plus trial. *β*-Coefficients and 95 % confidence intervals (total population = 3860)

Total DDS	Q1 (*n* 908)		Q2 (*n* 947)		Q3 (*n* 984)		Q4 (*n* 1021)	
	*β*	95 % CI	*β*	95 % CI	*β*	95 % CI	*β*	95 % CI
Model 1	0	Ref.	−0·04	−0·47, 0·38	0·03	−0·40, 0·45	0·12	−0·30, 0·55
Model 2	0	Ref.	0·02	−0·41, 0·44	0·08	−0·35, 0·50	0·22	−0·21, 0·65
Vegetable group	C1 (*n* 308)		C2 (*n* 713)		C3 (*n* 1430)		C4 (*n* 1409)	
Model 1	0	Ref.	0·59	−0·10, 1·28	0·71	−0·07, 1·35	0·57	−0·07, 1·21
Model 2	0	Ref.	0·62	−0·07, 1·32	0·72	0·07, 1·37	0·70	0·05, 1·35
Fruit group	C1 (*n* 475)		C2 (*n* 2611)		C3 (*n* 482)		C4 (*n* 292)	
Model 1	0	Ref.	0·18	−0·32, 0·69	−0·08	−0·73, 0·58	−0·38	−1·13, 0·37
Model 2	0	Ref.	0·12	−0·39, 0·64	−0·11	−0·78, 0·56	−0·35	−1·12, 0·42
Cereal group	C1 (*n* 173)		C2 (*n* 2774)		C3 (*n* 860)		C4 (*n* 53)	
Model 1	0	Ref.	−0·55	−1·34, 0·24	−0·38	−1·22, 0·46	−0·63	−2·21, 0·96
Model 2	0	Ref.	−0·52	−1·33, 0·29	−0·31	−1·19, 0·57	−0·61	−2·26, 1·04
Proteins group	C1 (*n* 8)		C2 (*n* 638)		C3 (*n* 1614)		C4 (*n* 1600)	
Model 1	0	Ref.	−2·13	−5·72, 1·45	−2·09	−5·67, 1·48	−2·52	−6·10, 1·05
Model 2	0	Ref.	−2·30	−5·88, 1·28	−2·26	−5·83, 1·30	−2·63	−6·20, 0·94
Dairy group	C1 (*n* 397)		C2 (*n* 1442)		C3 (*n* 1510)		C4 (*n* 511)	
Model 1	0	Ref.	−0·43	−1·00, 0·14	−0·27	−0·85, 0·30	−0·52	−1·20, 0·16
Model 2	0	Ref.	−0·39	−0·97, 0·19	−0·21	−0·80, 0·38	−0·45	−1·15, 0·25

DDS, dietary diversity score; Q, quartile (Q1, less diversity; Q4, more diversity).

Values are presented as *β*-coefficients and 95 % CI for changes in depressive symptomatology after 2 years of follow-up as continuous variable according to total DDS.

Model 1: Adjusted for sex and age.

Model 2: Additionally adjusted for depressive symptomatology at baseline, smoking habits, physical activity, educational level, BMI, living alone, civil status, sleep duration, presence of chronic diseases, allocation group and recruitment centre.

Values presented in bald showed a statistically significant association (*P* < 0·05).

DDS cut-off points for each quartile: (Q1 = 0·8–3·9, Q2 = 4·0–4·6, Q3 = 4·7–5·4 and Q4 = 5·5–8·0).

The variety in each food group was classified into four categories (C): (C1 = 0 points), (C2 => 0–≤0·5 points), (C3 => 0·5–<1 points) and (C4 ≥ 1 point).

Considering that the allocation group could exert an interaction with DDS and/or variety in food in depression, we explored this fact in the multivariate analysis. This variable was not an ‘effect modifier’ in the association between the changes in depressive symptomatology and DDS/food groups (*P*_for interaction_ >0·05) (data not shown). In order to avoid that the exclusion of individuals with high baseline depressive symptomology or with prevalent depression at baseline limits the possibility of finding longitudinal associations, we performed an ancillary analyses, not excluding those subjects with a Beck-II punctuation higher than 18 points at baseline or with prevalent depression at baseline. In the subsample analysed, the results obtained were not significant; however, the magnitude of the effect observed was quite similar to that found in the overall sample.

## Discussion

The present analysis was conducted as an observational prospective cohort study within the PREDIMED-Plus trial. In the cross-sectional analysis, total DDS was inversely associated with prevalent depression. Thus, study participants with higher DD (Q4) showed a significant decrease in the odds of depression compared to participants with lower DD (Q1). Taking into account each of the components of the total DDS, the consumption of a high diversity of vegetables, cereals and proteins also showed an inverse association with prevalence of depression in cross-sectional analyses. Nevertheless, in the longitudinal analysis, after 2 years of follow-up we did not find any significant association, except for the vegetable group, which, unexpectedly showed a positive association with an increasing risk of depressive symptomatology over time.

Some authors have pointed out that monotonous and unhealthy dietary patterns are directly associated with a higher risk of depression in community-dwelling adults^(36)^. According to our cross-sectional results, this study primarily showed that the variety of some food’s groups is related to lower prevalence of depression, particularly for vegetables, cereals and proteins diversity. A possible explanation for this finding could be that these food groups have a specific role against oxidative stress and brain signalling which could contribute to reduce depression in adults^(36)^. Particularly, the beneficial role of dietary fibre (main component of some food groups as vegetables, fruits and whole cereals) in the prevention of depressive disorders maybe related with gut microbiota composition and activity, including some mechanisms linked with the gut–bran axis, immune, neural and metabolic pathways involved in depression^(37,38)^. For instance, whole grains and vegetables are rich sources of fibre, antioxidant vitamins and flavonoids; meanwhile, protein food (fish and seafood, white meat, legumes, nuts and eggs) contains folate and B-vitamins. Furthermore, these food groups are important components of the Mediterranean diet, which has been extensively reported with lower likelihood of depressive symptoms in older adults^(39,40)^.

In nutritional epidemiology, dietary pattern analysis has emerged as an alternative and complementary approach to examining the relationship between diet and the risk of chronic diseases. Instead of looking at individual nutrients or foods, pattern analysis examines the effects of overall diet^(41)^. This approach is able to assess the overall food pattern because it goes beyond nutrients or foods and examines the effects of the overall diet, capturing a wide range of potential interactions between different nutrients and foods^(41)^. According to this concept, we constructed a DDS originally developed by Kant *et al.* ^(26)^ that reflects the diversity of food and provides greater knowledge about the dietary pattern in an objective way.

Our cross-sectional results showed that total DDS had an inverse association with depression at baseline. Participants in the highest DDS quartile showed a significantly lower depression prevalence compared to those participants in the lowest quartile. The results of the present study are in line with previous studies which employed self-reported questionnaire to evaluate depressive symptomatology that have shown the same trend in a cohort of Chinese pregnant women^(15)^ in a cohort of a Japanese community-dwelling aged 65 years or older^(42)^ and also, in the PREDIMED-Plus cohort^(43)^. This association could be related to the fact that a dietary pattern which contains more healthy food sources of major nutrients, such as vitamins and minerals, would decrease the risk of depression given that nutrients may affect brain development and functioning as we mentioned previously^(44,45)^.

However, we have to highlight the fact that the reported analyses are cross-sectional. In this sense, a cross-sectional study does not provide the temporal relationship between food intake and depression. That is, nutrition could play an important role in the development, course and treatment of depression, but at the same time depressive symptoms might also predict the adoption of poor diet (‘reverse causality’)^(46)^. In fact, some authors have pointed out that depressed individuals tend to have unhealthy behaviours such as engaging in less physical activity and poor dietary habits^(47)^. Either way, recent meta-analyses have indicated that dietary interventions based on adherence to healthy dietary patterns produce not only a reduction in depressive symptoms but also a lower risk of developing depressive symptoms in non-clinical populations^(48)^.

Although an inverse association was observed in cross-sectional analyses, we did not find any statistically significant association between total DDS (or the variety of food groups) and depressive symptomatology after 2 years of follow-up, except for the variety of vegetable food group. Although some prospective studies have pointed out that the intakes of some food groups, fundamentally fruits and vegetables and protein food groups (meat and fish), are protective against (incident) depression and depressive symptoms in non-European elderly populations^(49)^, several methodological aspects such as the use of different questionnaires, the measure of total intake instead of DD, the disease induction time or the brevity in the follow-up period could explain the differences observed between our study and other published analyses. In line, with our longitudinal findings, the MooDFOOD randomised clinical trial reported that among overweight or obese adults with subsyndromal depressive symptoms and multinutrient supplementation compared with placebo did not reduce episodes of major depressive disorder during 1 year^(50)^.

The current study has some limitations that should be noted. First, the results cannot be extrapolated to other populations, as the PREDIMED-Plus study population (participants with overweight or obesity and MetS) is not representative of the general population; however, our study population represents a significant proportion of current Western societies. Second, although the FFQ is a nutritional validated tool^(23)^, self-reporting questionnaires, in combination with memory loss of older participants, might lead a no differential misclassification bias. Nevertheless, this bias would tend to the null value, so the association would be greater than observed. Moreover, we excluded participants with energy intakes outside of predefined limits proposed by Willet *et al.* ^(22)^ using in addition the residual method in order to adjust for energy intake. Third, the DDS is a simple count of food groups consumed developed as indicator of nutritional adequacy that excludes non-recommended food products that are high in sugar, saturated fatty acids and meats owing to the high-energy density of these foods, as well as their low-nutrient density with high levels of Na, sugar and saturated fat. Thus, we considered that any intake of these not recommended food products would not increase DD. Despite this, we have not distinguished the subgroups foods following the original categorisation proposed by other authors^(17,26)^. We have previously shown that this score which evaluates DD is correlated to better micronutrient intake and overall dietary quality in the Spanish older adult population^(11,18)^.

Fourth, a selection bias may be present, since after 2 years of follow-up, only the healthiest participants would remain in the longitudinal study, producing an attenuation of the association found. Furthermore, significant associations were found only in cross-sectional analysis, but not in longitudinal, so we cannot elucidate a possible reverse causality. Finally, the follow-up time considered (only 2 years) is probably too short to assess changes in the primary outcome.

However, our study presents several strengths that enhance our findings. We used a repeated and validated measurement of the outcome over 2 years. Another strength is that, besides the use of a DDS that provides a more intuitive view of the whole dietary pattern, we also examined the variety of each food group, which allowed us to identify some of them as important components linked to depression. Another strength is the large sample size with a multicentre design and a longitudinal approach. Finally, the considerable amount of participant information collected using a standardised protocol that reduces information bias regarding reported food intakes, sociodemographic characteristics and lifestyle variables are other strengths that should be taken into account.

Our results suggest that recommending diets with high diversity of vegetables, grains and protein food groups (fish/seafood, white meat, nuts, eggs and legumes) may represent an effective approach to improve depression outcomes in community-dwelling population with overweight/obesity and MetS. That is, in people with depressive symptoms fostering dietary patterns such as the MedDiet would presumably result in a far greater impact over prevalence and symptomatology on depression. Nevertheless, these associations were only found in cross-sectional analysis. It is necessary to assess the entire cohort for longer in order to establish significant associations between DD and depression status.

In summary, our study found that higher DDS, and in particular, a high diversity intake of vegetables, cereals and proteins (fish/seafood, legumes, nuts, eggs and white meat) was inversely associated with depression status at baseline in community-dwelling older Spanish people. However, these result did not replicate in the longitudinal analysis. For that reason, further longitudinal studies with longer follow-up are needed to confirm our findings and deepen the understanding about the relationship between DD and depression status.
